# Mechanical Loading Reduces Inflammation-Induced Human Osteocyte-to-Osteoclast Communication

**DOI:** 10.1007/s00223-015-9999-z

**Published:** 2015-05-13

**Authors:** Janak L. Pathak, N. Bravenboer, Frank P. Luyten, Patrick Verschueren, Willem F. Lems, Jenneke Klein-Nulend, Astrid D. Bakker

**Affiliations:** Department of Oral Cell Biology, Academic Centre for Dentistry Amsterdam (ACTA), University of Amsterdam and VU University Amsterdam, MOVE Research Institute Amsterdam, Amsterdam, The Netherlands; Skeletal Biology and Engineering Research Center, KU Leuven, Leuven, Belgium; Department of Clinical Chemistry, VU University Medical Center, MOVE Research Institute Amsterdam, Amsterdam, The Netherlands; Department of Rheumatology, VU University Medical Center, MOVE Research Institute Amsterdam, Amsterdam, The Netherlands

**Keywords:** Rheumatoid arthritis, Generalized osteoporosis, Inflammatory cytokines, Pulsating fluid flow, Osteoclastogenesis

## Abstract

Multiple factors contribute to bone loss in inflammatory diseases such as rheumatoid arthritis (RA), but circulating inflammatory factors and immobilization play a crucial role. Mechanical loading prevents bone loss in the general population, but the effects of mechanical loading in patients with RA are less clear. Therefore, we aimed to investigate whether mechanical stimuli reverse the stimulatory effect of RA serum on osteocyte-to-osteoclast communication. Human primary osteocytes were pretreated with 10 % RA serum or healthy control serum for 7 days, followed by 1 h ± mechanical loading by pulsating fluid flow (PFF). Nitric oxide (NO) and prostaglandin E_2_ were measured in the medium. Receptor activator of nuclear factor-kappaB ligand (RANKL), osteoprotegerin (OPG), interleukin-6 (IL-6), cyclooxygenase-2 (COX2), matrix-extracellular phosphoglycoprotein (MEPE), cysteine-rich protein 61 (CYR61), and SOST gene expression was quantified by qPCR. Osteoclast precursors were cultured with PFF-conditioned medium (PFF-CM) or static-conditioned medium (stat-CM), and osteoclast formation was assessed. RA serum alone did not affect IL-6, CYR61, COX2, MEPE, or SOST gene expression in osteocytes. However, RA serum enhanced the RANKL/OPG expression ratio by 3.4-fold, while PFF nullified this effect. PFF enhanced NO production to the same extent in control serum (2.6–3.5-fold) and RA serum-pretreated (2.7–3.6-fold) osteocytes. Stat-CM from RA serum-pretreated osteocytes enhanced osteoclastogenesis compared with stat-CM from control serum-pretreated osteocytes, while PFF nullified this effect. In conclusion, RA serum, containing inflammatory factors, did not alter the intrinsic capacity of osteocytes to sense mechanical stimuli, but upregulated osteocyte-to-osteoclast communication. Mechanical loading nullified this upregulation, suggesting that mechanical stimuli could contribute to the prevention of osteoporosis in inflammatory disease.

## Introduction

Many patients with chronic inflammatory disease such as rheumatoid arthritis (RA) suffer from generalized osteoporosis [1, 2]. The cause of bone loss during inflammation is multifactorial and includes reduced physical activity, use of corticosteroids, and increased levels of inflammatory cytokines [2–4]. Altered levels of growth factors, growth factor antagonists, and inflammatory cytokines such as IL-1β, IL-6, and TNF-α are present in synovial fluid and serum from RA patients [5–7]. These factors present in RA serum easily reach the bone and affect the formation and function of osteoblasts, osteoclasts, and osteocytes as well as the communication between these bone cells [7–9]. Circulating inflammatory cytokines in patients with inflammatory disease likely play an important role in bone homeostasis, since individual cytokines such as IL-1β enhance osteocyte-mediated osteoclastogenesis [10], and serum from patients with active RA inhibits osteoblast proliferation and differentiation, and enhances osteoblast-mediated osteoclastogenesis [8]. Reduced physical activity is frequently observed in patients with RA, which is another important cause of bone loss in RA [11].

Cytokines and growth factors are not the only factors modulating bone homeostasis. During daily activities, bones are subjected to a variety of mechanical loads which affect bone remodeling and architecture. Osteocytes play a vital role in the adaptation of bone to mechanical loads since they translate mechanical stimuli into a biological response [12–14]. Osteocytes sense mechanical stimuli and produce signaling molecules that are potent regulators of the recruitment and activity of bone-forming osteoblasts, as well as bone-resorbing osteoclasts and their precursors [10, 15–17]. In response to mechanical stimuli, osteocytes release bone anabolic factors such as NO and PGE_2_ [18, 19]. Recently, it has been reported that mechanically loaded osteocytes produce IL-6, which affects both osteoclasts and osteoblasts [20]. Osteocytes release pro-osteoclastogenic signals in the absence of mechanical loading, leading to stimulation of bone resorption [21]. In the presence of mechanical stimuli, osteocytes produce factors that inhibit osteoclastogenesis, and/or decrease the production of osteoclast-stimulating signals [22, 23]. Mechanical stimuli affect the production of cytokines and signaling molecules such as IL-6, RANKL, OPG, CYR61, MEPE, COX-2, and sclerostin by osteocytes [23–25]. IL-6 and RANKL enhance osteoclastogenesis, while OPG, MEPE, and CYR61 inhibit osteoclastogenesis [23–26]. PGE2 enhances RANKL gene expression by osteoblasts [27]. Sclerostin may have a catabolic action through promotion of osteoclast formation and activity by osteocytes, in a RANKL-dependent manner [28].

Recombinant IL-1β and TNF-α reduce the physiological response of osteocytes to mechanical loading [29, 30], but whether the inflammatory factors as present in the serum of patients with active RA affect the response of osteocytes to mechanical stimuli is still unclear. Previously, it has also been shown that mechanical loading of MLO-Y4 osteocytes is perfectly able to reduce the stimulatory effect of recombinant IL-1β on osteocyte-to-osteoclast communication [10]. Whether mechanical loading is able to alter the stimulatory effect of RA serum on human osteocyte-to-osteoclast signaling, resulting in changes in bone resorption, is unknown. In this study, we tested the hypothesis that mechanical stimuli reverse the stimulatory effect of RA serum on osteocyte-to-osteoclast communication.

## Methods

### Recruitment of RA Patients

RA patients with active stage of disease were recruited (mean age: 62 ± 12 years; 6 females, 2 males), and diagnosed according to the 1987 RA classification at an early stage of the disease (less than 1 year disease duration) and before they had taken DMARDs or corticosteroids. Blood samples were collected, and within 1 h centrifuged for 10 min at 3000 rpm to separate the sera, that were aliquoted and stored at −80 °C. Patient characteristics, demographics, and clinical data (DAS score, Serum C-reactive protein (CRP)) were collected (Table 1). Blood samples were also collected from age- and gender-matched healthy controls. Patients with thyroid dysfunction, other inflammatory diseases besides RA, and pregnancy were excluded.

**Table 1:** Characteristics and demographics of the RA patients and healthy controls included in this study

	Active RA patients	Healthy controls
Sex (female/male)	6/2	6/2
Age (years)	62.3 ± 12.1	62.1 ± 12.4
DAS28 score	4.9 ± 1.3	n.d.
CRP (mg/l)	36.2 ± 40.3	<2.5
# RF-positive patients	2	n.d.

Values are mean ± SD. Eight active RA patients were included in this study

*DAS28 score* disease activity score, *CRP* C-reactive protein, *RF* rheumatoid factor, *n.d.* not determined

### Human Osteocyte Culture

Trabecular bone samples (surgical waste) from 2 male donors (age: 38 and 73 years) and 1 female donor (age: 55 years) were obtained from the anterior iliac crest during sinus floor elevation surgery using autologous anterior iliac crest bone graft. CRP levels of all donors was <2.5 mg/l, indicating no inflammatory disease in all donors.

Human primary bone cells were established as described earlier [30], and used as a model for osteocytes. Briefly, trabecular bone pieces were chopped into small fragments, and washed extensively with phosphate buffered saline (PBS). Bone fragments were then incubated with 2 mg/ml collagenase type II (Worthington, Freehold, NJ) in Dulbecco’s Modified Eagle’s Medium (DMEM; Gibco, Paisley, UK)/Nutrient mixture F-12 (F-12) (DMEM/F-12, 1:1 (v/v)) for 2 h at 37 °C in a shaking water bath to remove all adhering cells from the bone chip surfaces. Bone fragments were then washed with medium containing 10 % Fetal Clone I serum (HyClone, South Logan, UT), subdivided into equal portions, and transferred to 75 cm^2^ culture flasks (Greiner Bio-One, Kremsmuenster, Austria). To obtain outgrowth of bone cells, bone fragments were cultured in DMEM/F-12 supplemented with 10 % Fetal Clone I serum, 100 U/ml penicillin (Sigma, Hamburg, Germany), 100 µg/ml streptomycin sulfate (Gibco), 50 µg/ml gentamicin (Gibco), 1.25 µg/ml fungizone (Gibco), and 100 µg/ml ascorbate (Merck, Darmstadt, Germany) at 37 °C in a humidified atmosphere with 5 % CO_2_. The culture medium was refreshed twice a week. Cultures were continued till ~90 % confluency.

### Effect of Active RA Serum and Pulsating Fluid Flow (PFF) on Osteocyte Culture

Outgrowth bone cells between passages 1–3 were trypsinized with 0.25 % trypsin (Difco Laboratories, Detroit, MI) and 0.1 % EDTA (Sigma) in PBS. Cells were seeded at a density of 1 × 10^5^ cells/25 cm^2^ culture flask (Nunc, Roskilde, Denmark) and incubated overnight. Then cells were cultured in DMEM with 10 % RA serum or healthy control serum for 7 days to mimic chronic systemic inflammation during RA, and culture medium was refreshed after 3 days with medium also containing 10 % RA serum or healthy control serum. After 7 days, cells were trypsinized and seeded onto polylysine-coated (50 µg/ml poly-l-lysine hydrobromide, Sigma) glass slides (size 22 × 22 mm) at 5 × 10^4^ cells/glass slide, and cultured overnight in six well plates with DMEM containing 10 % RA serum or healthy control serum. The next day culture medium was replaced by DMEM with 0.2 % bovine serum albumin (BSA), and the cells were subjected to 1 h PFF as described previously [18, 19, 29, 31]. Briefly, cells were subjected to PFF (mean ± amplitude 0.7 ± 0.7 Pa, 5 Hz) by pumping 4 ml of culture medium with 0.2 % BSA through a parallel-plate flow chamber containing the osteocytes. Stationary control cultures were kept in six well plates under similar conditions as the experimental cultures, i.e., at 37 °C in a humidified atmosphere of 5 % CO_2_ in air. After 5 and 60 min of PFF or static culture, the medium was collected and assayed for NO and PGE_2_ production. After 1 h PFF, treatment was terminated, and the cells were post-incubated in fresh DMEM with 0.2 % BSA for 1 h without mechanical loading. After 1 h post-incubation, the CM was collected and cells were processed for total RNA isolation.

### NO and PGE_2_ Production

NO production was measured as nitrite (NO_2_^−^) accumulation in the CM using Griess reagent containing 1 % sulfanylamide, 0.1 % naphtylethelene-diamine-dihydrochloride, and 2.5 M H_3_PO_4_. Serial dilutions of NaNO_2_ in non-CM were used as a standard curve. Absorbance was measured at 540 nm with a microplate reader (Bio-Rad Laboratories). PGE_2_ was measured by using PGE_2_ High Sensitivity ELISA Kit (Abcam^®^, Cambridge, UK).

### RNA Isolation and Real-Time RT-PCR

Total RNA from osteocytes was isolated using an RNeasy^®^ Micro kit with an on-column DNase I digestion (Qiagen, Basel, Switzerland). Total RNA concentrations were measured with a Nanodrop spectrophotometer (Nanodrop Technologies, Wilmington, DE). cDNA synthesis was performed in a thermocycler GeneAmp^®^ PCR System 9700 PE (Applied Biosystems, Foster City, CA), using aSuperScript^®^ VILO™ cDNA Synthesis Kit (LifeTechnologies, Inchinnan, UK), with 0.1 μg of total RNA in 20 μl reaction mixture consisting of VILO Reaction Mix and SuperScript Enzyme Mix. cDNA was stored at −20 °C until real-time PCR analysis. Real-time PCR reactions were performed using 2.0 μl cDNA and SYBR^®^ Green Supermix (Roche Laboratories, Indianapolis, IN) in a LightCycler^®^ (Roche Diagnostics, Switzerland). In each PCR run, the reaction mixture without cDNA was used as a negative control. For quantitative real-time PCR, the values of relative target gene expression were normalized to relative housekeeping gene (YWHAZ) expression. Real-time PCR was used to assess expression of the following genes: COX-2, RANKL, OPG, MEPE, SOST, and IL-6. All primers used for real-time PCR were from Life Technologies. The primer sequences are listed at Table 2. In each assay, for osteogenic marker gene expression, mRNA preparations of human bone were used as a reference and internal control for the primer sets to pick up the specific mRNA of interest.

**Table 2:** Primers used in the real-time PCR assay

Gene	Oligonucleotide sequence		Amplicon length (bp)
YWHAZ	Forward	5′ GATGAAGCCATTGCTGAACTTG 3′	229
	Reverse	5′ CTATTTGTGGGACAGCATGGA 3′	
COX2	Forward	5′ GCATTCTTTGCCCAGCACTT 3′	299
	Reverse	5′ AGACCAGGCACCAGACCAAAGA 3′	
CYR61	Forward	5′ CAACCCTTTACAAGGCCAGA 3′	206
	Reverse	5′ TGGTCTTGCTGCATTTCTTG 3′	
IL-6	Forward	5′ ACAGCCACTCACCTCTTCA 3′	207
	Reverse	5′ ACCAGGCAAGTCTCCTCAT 3′	
OPG	Forward	5′ TGGAATAGATGTTACCCTGTGTG 3′	298
	Reverse	5′ GCTGCTCGAAGGTGAGGTTA 3′	
RANKL	Forward	5′ CATCCCATCTGGTTCCCATAA 3′	60
	Reverse	5′ GCCCAACCCCGATCATG 3′	
SOST	Forward	5′ GGGTGGCAGGCGTTCA 3′	164
	Reverse	5′ CTGTACTCGGACACGTCTTTGGT 3′	
MEPE	Forward	5′ GAGTTTTCTGTGTGGGACTACTCCTT 3′	101
	Reverse	5′ TCTGCTCTTCCACACAGCTTTG 3′	

### Osteoclastogenesis

Peripheral blood mononuclear cells (PBMCs) were isolated from a buffy coat (Sanquin, Amsterdam, The Netherlands) as described previously [32]. PBMCs were seeded at 5 × 10^5^ cells/well of 96-well plates in DMEM containing 10 % FCS, antibiotics (100 U/ml penicillin, 100 g/ml streptomycin, and 250 ng/ml amphotericin B), and control serum-pretreated CM (static and PFF), or RA serum-pretreated CM (static and PFF) (ratio DMEM:CM = 1:1 (v/v)). Twenty-five ng/ml recombinant human M-CSF (R&D Systems, Minneapolis, MN) was added to the cells from day 1 to day 3. Ten ng/ml M-CSF and 4 ng/ml human RANKL (Peprotech, London, UK) were added from day 3 to day 21. After 3 weeks of culture, cells were fixed in 4 % formaldehyde and stained for tartrate-resistant acid phosphatase (TRACP; TRAP Kit, Sigma). Nuclei were visualized by 4′,6-diamidino-2-phenylindole (DAPI) staining. Osteoclast formation was assessed by counting the number of TRACP-positive multinucleated cells (MNCs), containing 3 or more nuclei per cell. Osteoclasts were counted in five fixed microscopic fields of each well using a Leica DM IL microscope (Leica, Wetzlar, Germany) equipped with a 20× objective.

### Statistical Analysis

Data are expressed as mean. The effects of RA serum or PFF on NO and PGE_2_ production, gene expression of cytokines and growth factors, and osteoblast-mediated osteoclastogenesis were tested by one-way analysis of variance (ANOVA). ANOVA was applied to the four groups for each parameter analyzed, followed by Bonferroni’s multiple comparison test as post hoc test. Differences were considered significant if *p* < 0.05. Statistical analysis was performed using GraphPad Prism 5.01 (GraphPad Software, Inc., La Jolla, CA, USA).

## Results

### RA Serum Enhanced Osteoclastogenic Gene Expression by Osteocytes, While PFF Attenuated this Effect

**Fig. 1:**
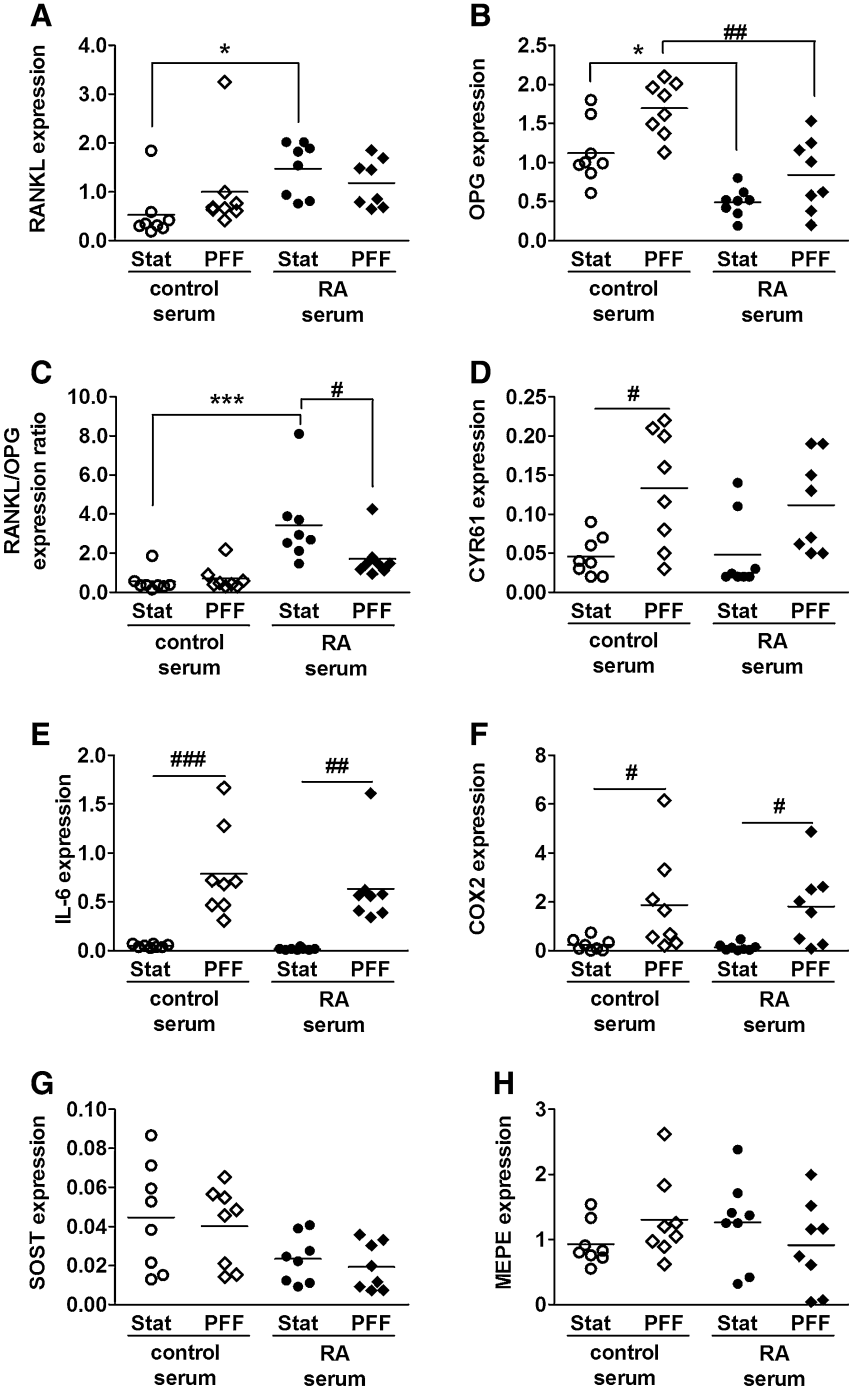
Effect of RA serum and/or PFF treatment on cytokine and growth factor/inhibitor gene expression by primary osteocytes. Cells were cultured for 7 days with or without RA serum, followed by 1 h PFF or static control culture, and 1 h post-incubation without PFF. **a** RA serum enhanced RANKL gene expression. **b** RA serum inhibited OPG gene expression. It also reduced the stimulatory effect of PFF on OPG gene expression. **c** RA serum enhanced RANKL/OPG gene expression ratio, and PFF nullified this effect. **d** PFF enhanced CYR61 gene expression in control and RA serum-pretreated osteocytes. **e** PFF enhanced IL-6 gene expression in control and RA serum-pretreated osteocytes. **f** PFF enhanced COX2 gene expression in control and RA serum-pretreated osteocytes. **g** RA serum and/or PFF did not affect SOST gene expression, nor (**h**) MEPE gene expression. Values are mean from eight independent experiments. Significant effect of RA serum, *p < 0.05, ***p < 0.001. Significant effect of PFF, ^#^ *p* < 0.05, ^##^ *p* < 0.01, ^###^ *p* < 0.001. *Stat* static control culture, *PFF* pulsating fluid flow

We analyzed the effect of RA serum as well as the combination of RA serum and PFF on osteoclastic gene expression by primary osteocytes. Cells were subjected to PFF after 7 days of culture with RA serum or control serum. RA serum enhanced RANKL gene expression by twofold in static osteocytes (Fig. 1a). It inhibited OPG gene expression by 2.7-fold in static osteocytes and 2.8-fold in PFF-subjected osteocytes (Fig. 1b). RA serum enhanced the RANKL/OPG ratio by 3.4-fold in static osteocytes, while PFF treatment of RA serum-pretreated osteocytes nullified this effect (Fig. 1c). PFF enhanced CYR61 gene expression by 2.7-fold in control serum-pretreated osteocytes, and 2.3-fold in RA serum-pretreated osteocytes (Fig. 1d). PFF upregulated IL-6 gene expression in control osteocytes by 28-fold, and in RA serum-pretreated osteocytes by 33-fold (Fig. 1e). PFF upregulated COX2 gene expression in control osteocytes by 6.4-fold, and in RA serum-pretreated osteocytes by tenfold (Fig. 1f). SOST and MEPE gene expression were not affected by RA serum nor by PFF treatment (Fig. 1g, h).

### PFF Enhanced NO but not PGE_2_ Production in the Presence of RA Serum

**Fig. 2:**
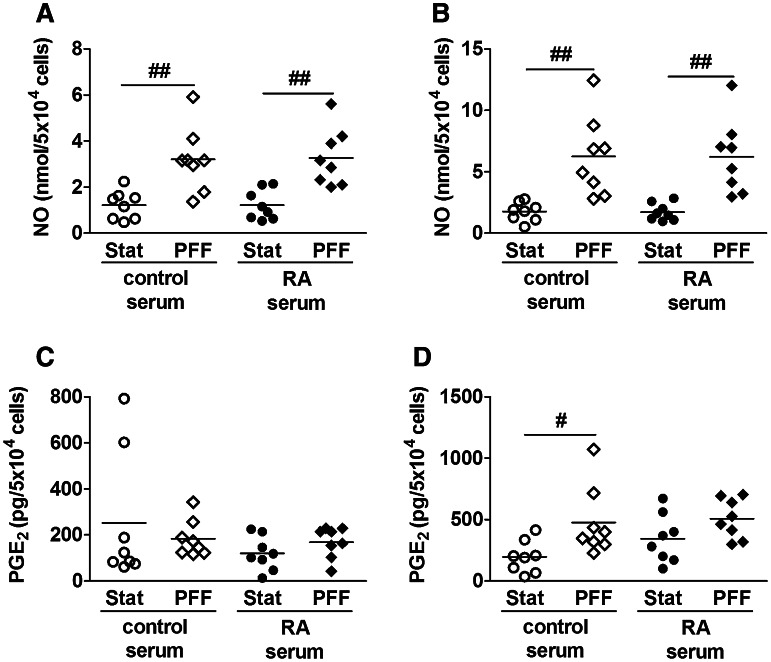
Effect of PFF on NO and PGE_2_ production by control serum and RA serum-pretreated primary osteocytes. **a** PFF enhanced NO production by control serum and RA serum-pretreated osteocytes at 5 min, and **b** at 60 min. **c** PFF did not affect PGE_2_ production in control serum or RA serum-pretreated osteocytes at 5 min. **d** PFF enhanced PGE_2_ production in control serum, but not RA serum-pretreated osteocytes at 60 min. Values are mean from eight independent experiments. Significant effect of PFF, ^#^ *p* < 0.05, ^##^ *p* < 0.01

PFF enhanced NO production by 2.6-fold at 5 min (Fig. 2a), and by 3.5-fold at 60 min (Fig. 2b) in control serum-treated osteocytes. Similarly, PFF treatment of RA serum-pretreated osteocytes enhanced NO production by 2.7-fold at 5 min (Fig. 2a), and by 3.6-fold at 60 min (Fig. 2b). RA serum pretreatment alone did not affect NO production. RA serum pretreatment and the combination of RA serum and PFF treatment did not affect PGE_2_ production by osteocytes at 5 min (Fig. 2c). PFF enhanced PGE_2_ production by 2.5-fold in control serum-pretreated osteocytes at 60 min, but not in RA serum-pretreated osteocytes (Fig. 2d).

### RA Serum-Pretreated Osteocytes Enhanced Osteoclast Formation, While PFF Nullified this Effect

**Fig. 3:**
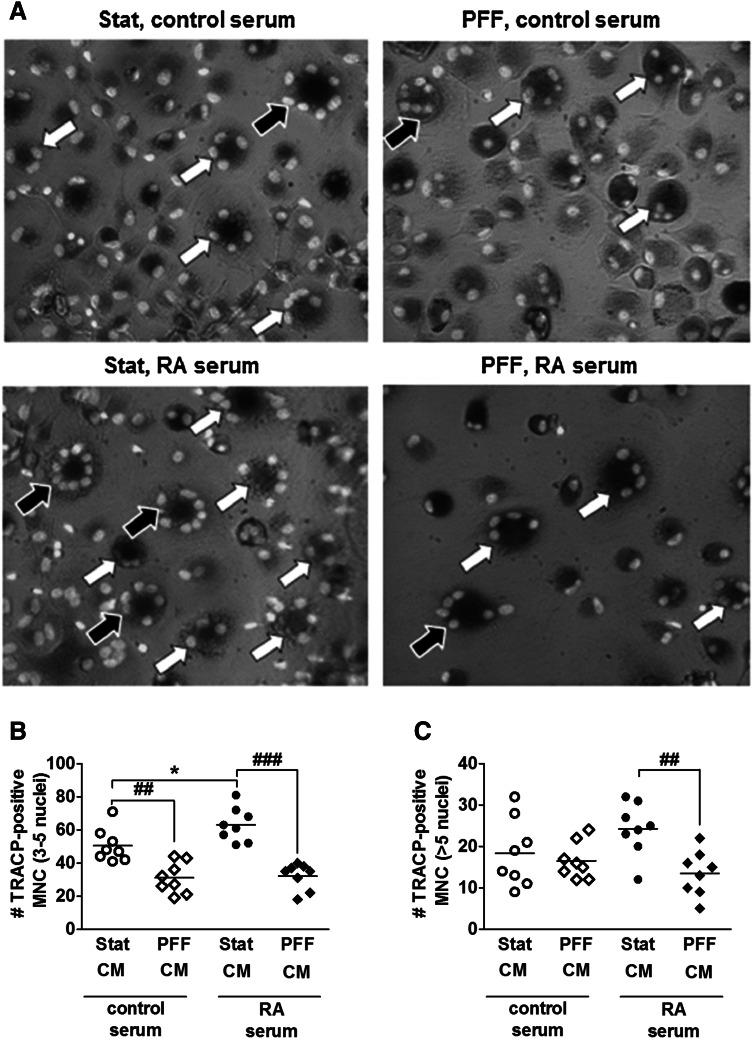
Effect of Stat-CM from RA serum-pretreated osteocytes and PFF-CM from RA serum-pretreated osteocytes on osteoclast formation. CM was obtained from osteocytes cultured for 7 days with or without RA serum, followed by 1 h PFF or static control culture, and 1 h post-incubation without PFF. CM was added to osteoclast precursors. **a** Representative micrograph of TRACP-positive multinucleated cells (TRAP + MNC) in a culture of human PBMCs with stat-CM from control serum-pretreated osteocytes, PFF-CM from control serum-pretreated osteocytes, stat-CM from RA serum-pretreated osteocytes, and PFF-CM from RA serum-pretreated osteocytes. *White arrows*: TRACP + MNC with 3–5 nuclei; black arrows: TRACP + MNC with >5 nuclei. **b** PFF-CM from control serum-pretreated osteocytes inhibited the formation of osteoclasts with 3–5 nuclei. Stat-CM from RA serum-pretreated osteocytes enhanced the formation of osteoclasts with 3–5 nuclei, and PFF-CM from RA serum-pretreated osteocytes nullified this effect. **c** PFF-CM from RA serum-pretreated osteocytes inhibited the formation of osteoclasts with >5 nuclei. Values are mean from three independent experiments, *n* = 9. Significant effect of Stat-CM from RA serum-pretreated osteocytes, *p < 0.05. Significant effect of PFF-CM, ^##^ *p* < 0.01, ^###^ *p* < 0.001

PFF-CM from control serum-pretreated osteocytes decreased the number of TRACP-positive osteoclasts with 3–5 nuclei by 1.6-fold (Fig. 3a, b). Stat-CM from RA serum-pretreated cell culture increased the number of TRACP-positive osteoclasts with 3–5 nuclei by 1.2-fold, while PFF nullified this effect (Fig. 3a, b). PFF-CM from RA serum-pretreated osteocytes also inhibited formation of osteoclasts with >5 nuclei by 1.8-fold (Fig. 3a, c).

## Discussion

Systemic inflammation and immobilization are associated with bone loss in RA [11]. In this study, we tested the hypothesis that mechanical stimuli reverse the stimulatory effect of RA serum on osteocyte-to-osteoclast communication. We found that RA serum did not affect the *intrinsic* capacity of osteocytes to sense mechanical stimuli, i.e., the osteocytes showed an unchanged NO response to PFF, although PGE_2_ production was affected, and an unaltered gene expression of IL-6, COX2, CYR61, MEPE, and SOST. RA serum upregulated osteocyte-to-osteoclast communication and osteocyte-mediated osteoclastogenesis. Interestingly, pulsating fluid flow attenuated the stimulatory effect of RA serum on osteocyte-to-osteoclast communication and osteocyte-mediated osteoclastogenesis. Our data indicate that mechanical stimuli on osteocytes may prevent inflammation-driven osteocyte-mediated osteoclastogenesis in RA.

We found that RA serum enhanced the gene expression ratio of RANKL/OPG in primary osteocytes. Moreover, CM from RA serum-pretreated osteocytes enhanced osteoclast formation. These findings are in accordance with our previous findings showing that RA serum-pretreated human primary osteoblasts enhance osteoclastogenesis [8]. Kulkarni and colleagues showed that IL-1β not only enhances the gene expression ratio of RANKL/OPG in osteocytes, but it also stimulates osteocyte-mediated osteoclastogenesis in a dose-dependent manner [10]. In this study, we found that RA serum did not affect IL-6, CYR61, COX2, SOST, or MEPE gene expression, even though we previously found that RA serum enhances IL-6 gene expression in primary human osteoblasts [8]. This contradiction between current findings and earlier observations might be time-point related, i.e., in this study, we analyzed IL-6 gene expression after 7 days of RA serum treatment, while in our previous study IL-6 expression was assessed after 10 days of treatment with RA serum from a different RA patient group. Our data indicate that RA serum containing inflammatory cytokines enhances osteocyte-to-osteoclast communication, which may contribute to generalized osteoporosis in RA.

**Fig. 4:**
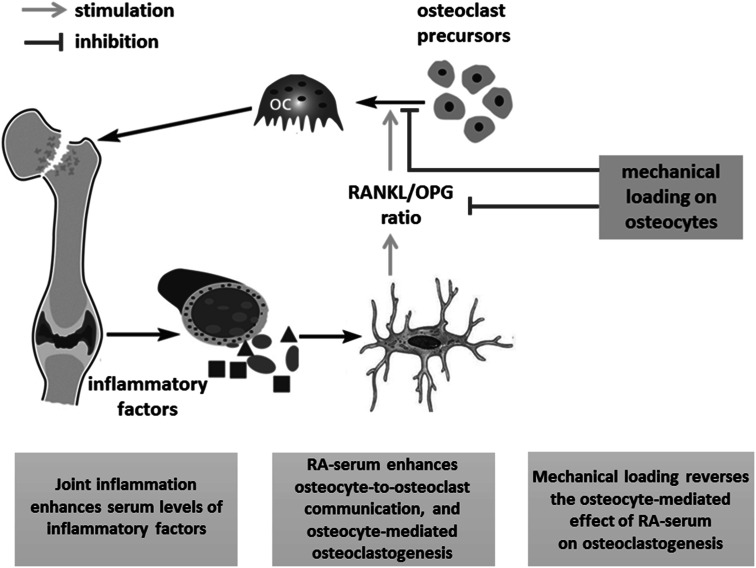
Pathophysiological model illustrating how mechanical loading can prevent inflammation-induced bone loss in RA. Whether mechanical loading-stimulated osteocytes produced factors that inhibit osteoclast activity was not tested in this study

Inflammatory cytokines such as IL-1β and TNF-α have been reported to reduce the mechanosensitivity of mouse osteocytes [29]. We found that RA serum did not affect the mechanosensitivity of human osteocytes, which might be explained by the multitude of factors present in RA serum, such as growth factors and their antagonists, pro-inflammatory as well as anti-inflammatory cytokines, cytokine receptors, and antibodies to cytokines. The combined effect of all these factors on the response of osteocytes to mechanical loading might be different from the effect of an individual recombinant cytokine. PFF nullified the stimulatory effect of RA serum on the RANKL/OPG expression ratio in osteocytes. Similarly PFF treatment of RA serum-pretreated osteocytes nullified the stimulatory effect of RA serum on bone cell-mediated osteoclastogenesis. Our findings are in accordance with data obtained by Kulkarni and colleagues showing that IL-1β enhances osteocyte-mediated osteoclastogenesis, and that mechanical loading reduces this effect [10]. We found that PFF enhanced IL-6, COX2, and CYR61 gene expression to a similar extent in RA serum- and control serum-treated osteocyte cultures, but it did not affect SOST and MEPE gene expression. Kulkarni and colleagues showed that PFF enhances MEPE expression in a murine osteocyte cell line (MLO-Y4) [23], while Robling and colleagues reported that high strain mechanical loading reduces sclerostin levels in mouse ulnae in vivo [25, 33]. Mechanical loading applied via oscillatory fluid flow for 2 h, but not 1 h, reduces SOST expression in UMR 106.01 osteoblasts [34]. This discrepancy might be due to differences in the cell types used, the magnitude and the type of loading applied, and the experimental set up such as in vitro 2D culture and an in vivo mouse model. We applied a peak shear stress of only 0.7 Pa for 1 h, while Papanicolaou and colleagues applied a peak shear stress of 2.0 Pa for 2 h on UMR 106.01 osteoblasts resulting in reduced SOST gene expression [34]. Moreover, we applied pulsating fluid flow on osteocytes for only 1 h, while Robling and colleagues loaded mice ulna in vivo by 360 cycles/day for two consecutive days [25]. In our study, the duration of mechanical loading and/or applied peak shear stress might not have been enough to affect SOST gene expression. We found that PFF did not inhibit the RANKL/OPG gene expression ratio in osteocytes treated with control serum. The RANKL/OPG pathway is important for osteoclastogenesis, but many other osteoclastogenesis-modulating signaling molecules are produced by osteocytes as well [23, 26]. In this study, we observed enhanced gene expression of CYR61 by osteocytes treated with control serum, which might explain the decreased osteoclastogenesis in cultures of osteocyte precursors with PFF-CM from control serum-pretreated osteocytes, since CYR61 inhibits osteoclastogenesis [26]. We found that PFF reduced both primary osteocyte-mediated osteoclastogenesis and the stimulatory effect of RA serum on primary osteocyte-mediated osteoclastogenesis. The effect of rheumatoid factor-positive and negative sera on cytokine and growth factor gene expression by osteocytes and on osteocyte-mediated osteoclastogenesis was similar. Our findings suggest an important role of mechanical loading in the inhibition of both physiological as well as inflammation-induced osteoclastogenesis. Based on our findings, we created a pathophysiological model illustrating how mechanical loading might prevent inflammation-induced bone loss in RA (Fig. 4).

A strength of our study is that we used a well-defined RA patient group as well as a well-defined healthy control group. We also used a well-established mechanical loading system applying pulsating fluid flow resulting in fluid shear stress on a monolayer of cells. In addition, we used well-established cell types, such as human primary osteocytes and human PBMCs. Our human primary osteocyte model is using primary bone cells, which have been shown to exhibit both an osteocyte and osteoblast-like phenotype [18, 31, 35, 36]. These cells are more close to the in vivo human bone niche in comparison to human or mouse osteoblast cell lines or primary mouse or chicken osteocytes. The relatively high expression of the osteocyte-specific genes MEPE and SOST compared to the housekeeping gene indicates the osteocytic nature of the cells used in this study. Today there is no better option than using human primary bone cells as a model for human osteocytes. Here osteoclast precursors were cultured with a mixture of CM (containing 0.2 % BSA) obtained from osteocytes pretreated with RA serum or control serum, and fresh DMEM (containing 10 % fetal clone serum) (CM:DMEM, 1:1, v/v). Healthy control serum and RA serum contain antibodies and autoantibodies that reduce osteoclastogenesis by inhibiting cytokine and signaling molecule function [37, 38]. In this study, osteoclast precursors were never in contact with RA serum or healthy control serum, thereby eliminating a possible direct effect of antibodies and autoantibodies present in the RA serum or healthy control serum on osteoclastogenesis. A limitation of our study might be the relatively low number of patients included. Statistical significance between groups is not easily obtained due to the fairly large data variation, probably due to donor variation. Another limitation of our study might be possible differences in the potency of the serum as a result of differences in storage time of the serum samples. The potency of serum decreases with storage time, and therefore active RA serum stored for more than 1 year might have lost some of its potency more so than serum stored for a shorter time.

In conclusion, we found that RA serum did not alter the intrinsic capacity of osteocytes to sense mechanical stimuli, but upregulated osteocyte-to-osteoclast signaling, while mechanical loading nullified this effect. Our data suggest that mechanical loading of bone might prevent osteoclast-related bone loss in RA patients by reversing the stimulatory effect of RA serum on osteocyte-to-osteoclast signaling. Physical activity or other forms of bone loading, e.g., vibrating platforms, could thus have great therapeutic potential in the prevention of osteoporosis in RA and other inflammatory diseases.
